# Supplementary material for: A Global Screen for Assembly State Changes of the Mitotic Proteome by SEC-SWATH-MS
Source: Cell Syst. 2020 Feb 26;10(2):133–155.e6. doi: 10.1016/j.cels.2020.01.001 (PMC7042714; doi:10.1016/j.cels.2020.01.001)

Q9BVT8 | TMUB1_HUMAN | TMUB1 C7orf21 DULP HOPS SB144 UNQ763/PRO1555
Monomer MW [kDa]: 26.261 Monomer expected elution fraction: 52

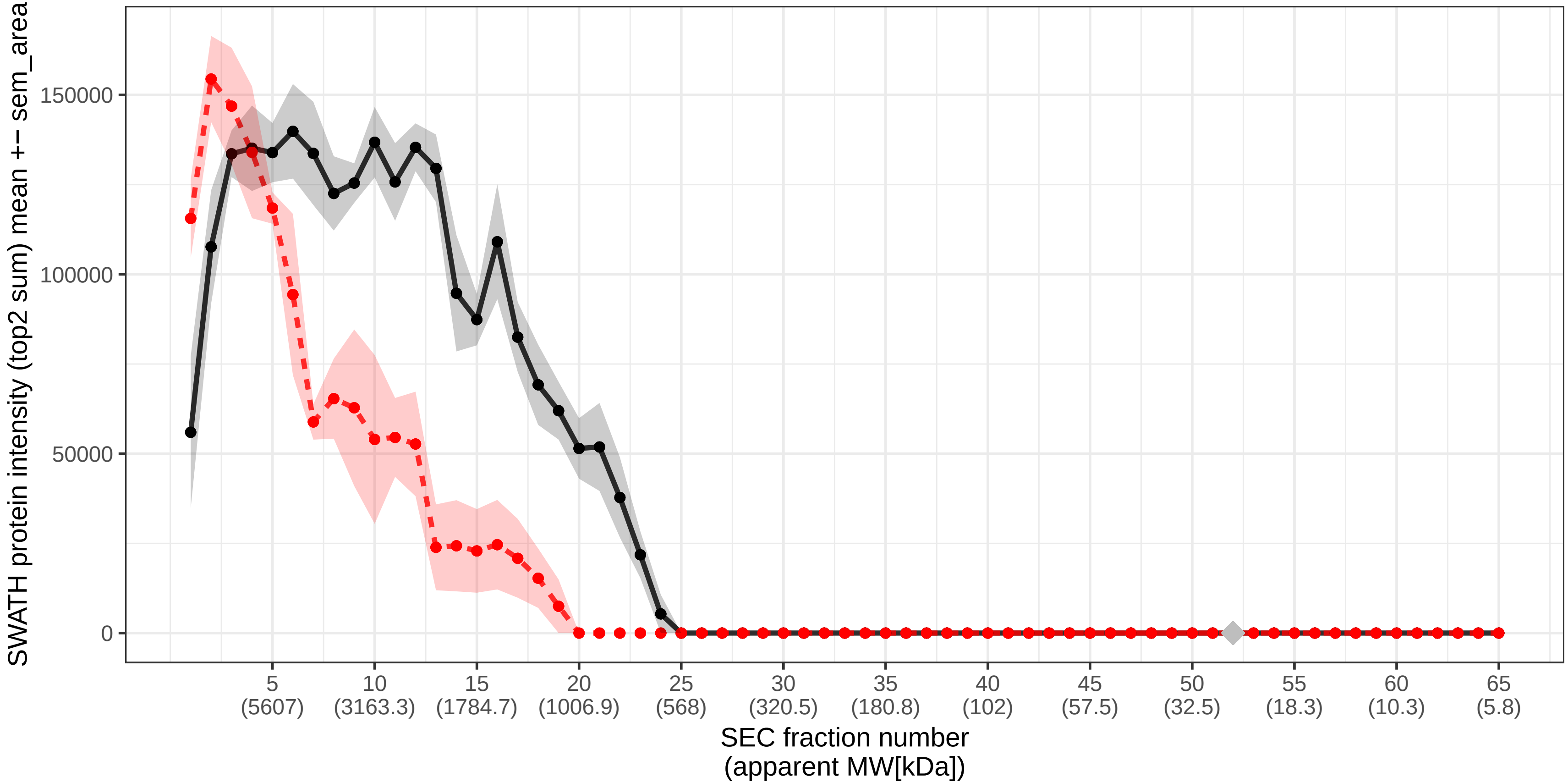

Supplement: Data S1. SEC-SWATH-MS Protein Chromatograms, Related to Figure 1 [file mmc6.zip › SECchrom_Q9BVT8_TMUB1_HUMAN_TMUB1_C7orf21_DULP_HOPS_SB144_U.pdf]
